# Spatial proteomics revealed a CX_3_CL1-dependent crosstalk between the urothelium and relocated macrophages through IL-6 during an acute bacterial infection in the urinary bladder

**DOI:** 10.1038/s41385-020-0269-7

**Published:** 2020-02-28

**Authors:** Jenny Bottek, Camille Soun, Julia K. Lill, Akanksha Dixit, Stephanie Thiebes, Anna-Lena Beerlage, Marius Horstmann, Annett Urbanek, Heike Heuer, Julian Uszkoreit, Martin Eisenacher, Thilo Bracht, Barbara Sitek, Franziska Hoffmann, Nirojah Vijitha, Ferdinand von Eggeling, Daniel R. Engel

**Affiliations:** 1 Institute of Experimental Immunology and Imaging, Department of Immunodynamics, University Hospital Essen, 45147 Essen, Germany; 2 Leibniz Institute of Photonic Technology, 07743 Jena, Germany; 3 Clinic for Endocrinology, University Hospital Essen, 45147 Essen, Germany; 4 Ruhr University Bochum, Medical Faculty, Medizinisches Proteom-Center, 44801 Bochum, Germany; 5 Department of Otorhinolaryngology, Jena University Hospital, 07743 Jena, Germany

## Abstract

The urothelium of the urinary bladder represents the first line of defense. However, uropathogenic *E. coli* (UPEC) damage the urothelium and cause acute bacterial infection. Here, we demonstrate the crosstalk between macrophages and the urothelium stimulating macrophage migration into the urothelium. Using spatial proteomics by MALDI-MSI and LC-MS/MS, a novel algorithm revealed the spatial activation and migration of macrophages. Analysis of the spatial proteome unravelled the coexpression of Myo9b and F4/80 in the infected urothelium, indicating that macrophages have entered the urothelium upon infection. Immunofluorescence microscopy additionally indicated that intraurothelial macrophages phagocytosed UPEC and eliminated neutrophils. Further analysis of the spatial proteome by MALDI-MSI showed strong expression of IL-6 in the urothelium and local inhibition of this molecule reduced macrophage migration into the urothelium and aggravated the infection. After IL-6 inhibition, the expression of matrix metalloproteinases and chemokines, such as CX_3_CL1 was reduced in the urothelium. Accordingly, macrophage migration into the urothelium was diminished in the absence of CX_3_CL1 signaling in *Cx*_*3*_*cr1*^*gfp/gfp*^ mice. Conclusively, this study describes the crosstalk between the infected urothelium and macrophages through IL-6-induced CX_3_CL1 expression. Such crosstalk facilitates the relocation of macrophages into the urothelium and reduces bacterial burden in the urinary bladder.

## Introduction

Acute bacterial infections in the urinary bladder are one of the most common and hospital-acquired infections with uropathogenic *Escherichia coli* (UPEC) as the main causative agent.^1,2^ These pathogens damage and invade into the urothelium of the urinary bladder,^3^ which accounts for the high amount of disease recurrence in patients. The local host inflammatory response in the urothelium is characterized by infiltration and transurothelial migration of neutrophils.^4^ Recent studies indicated that macrophages and urothelial cells recruit neutrophils into the infected urothelium by producing chemokines.^5–7^ The absence of macrophages impeded the response against UPEC.^8–10^ Furthermore, there is emerging evidence that macrophages retain free iron to limit UPEC growth^11^ and also phagocytose UPEC directly in an ATG16L1-dependent manner.^12,13^ However, the molecular mechanism which regulates the spatial distribution of macrophages within the urothelium upon UPEC infection has not been studied so far.

Imaging approaches, such as state-of-the-art microscopy and mass spectrometry imaging, are powerful technologies that gain spatial molecular information about the localization of proteins in tissues. Matrix-assisted laser desorption/ionization mass spectrometry imaging (MALDI-MSI) is widely used as a label-free imaging technique, providing important information on the proteomic landscape.^14,15^ It has the potential to unravel the molecular mechanisms that regulate the localization of leukocytes, such as macrophages.^16,17^ Technological and methodological advancements in the past 10–15 years have led to the refinement of this method with a broad range of applications.^18–20^ For proteomic applications,^21^ tissue samples are often subjected to an initial on-tissue tryptic digestion step, increasing mass resolution. In order to determine the identity of a specific *m/z* value, LC-MS/MS in combination with computational methods, existing libraries, and coregistration algorithms was used.^15,22,23^ Protein Inference Algorithm (PIA) has recently been established to combine peptide spectrum matches from different search engines, increasing consistency of the results.^24^ This study employs PIA in combination with computational and coregistration methods to establish the algorithm SPRING (Spatial PRoteome ImagiNG) that correlates mass spectrometry datasets and extracts spatial cellular and molecular information in biological samples. SPRING, in combination with state-of-the-art microscopy and experimental in vivo targeting approaches, unraveled the mechanism of macrophage relocation into the urothelium upon UPEC infection. We report here a novel IL-6-induced crosstalk between the urothelium and macrophages. This crosstalk facilitates a CX_3_CL1-dependent migration of macrophages into the urothelium to phagocytose UPEC. Conclusively, macrophage relocation maintains the barrier function of the urothelium and reduces bacterial burden in a model of acute bacterial infection of the urinary bladder.

## Results

### SPRING identifies macrophage-associated activity in the urothelium in a murine model of acute bacterial infection of the urinary bladder

**Fig. 1:**
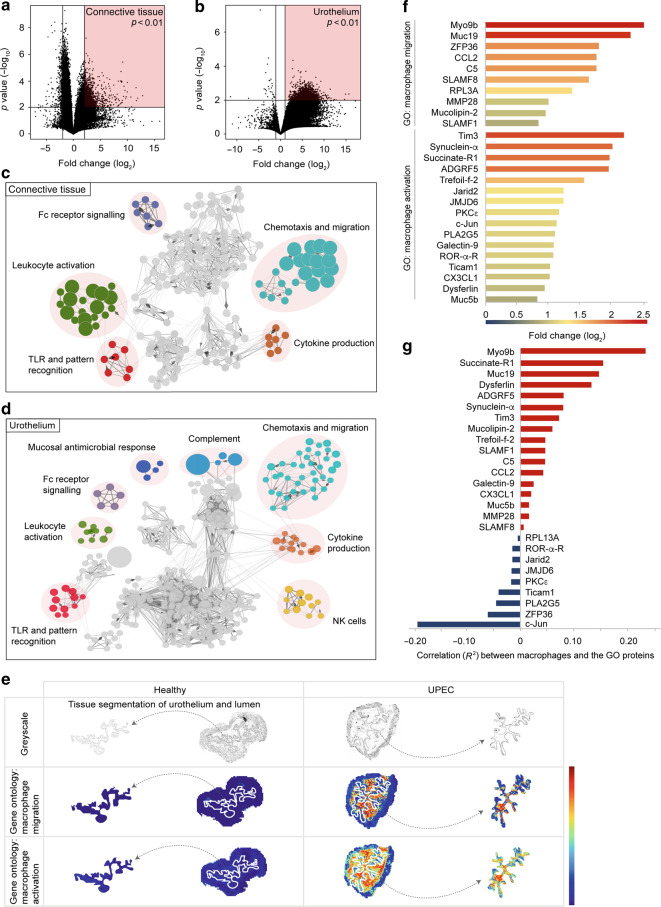
Mass spectrometry imaging indicates macrophage migration and activation within the infected urothelium. Mice were infected with UPEC and analyzed 1-day post-infection. **a**, **b** Volcano plots depicting the peptides from the MALDI spectrum upon UPEC infection, for both connective tissue (**a**) and urothelium (**b**). *P*-values were obtained with a Student *t*-test. The lines for the *p*-value and the fold change indicate a value of 0.01 and ±2, respectively. **c**, **d** Enrichment analysis by Cytoscape/ClueGO of the significantly *(p*-value < 0.01) upregulated (Fold-change > 2) proteins after infection. **e** Representative bladder tissue sections depicting the spatial distribution of the proteins of the murine GO terms “macrophage migration” (GO: 1905517) and “macrophage activation” (GO: 0042116) in healthy (left) and UPEC-infected (right) conditions by MALDI-MSI. The first row shows a greyscale image and the tissue segmentation of the urothelium from the urinary bladder. The second and the third row show the expression of the indicated GO terms. The white dashed lines separate the connective tissue from the urothelium and the lumen. The segmented urothelium and lumen are represented as individual images on the far right and far left. **f** Fold changes of the expression intensities of the proteins of the indicated GO terms in the urothelium in healthy versus UPEC-infected samples determined by MALDI-MSI (Formula = Log_2_(UPEC/Healthy)). **g** Correlation of the expression intensity of F4/80 and the proteins of the GO terms detected by MALDI-MSI. GO = gene ontology; Healthy: *n* = 3; UPEC: *n* = 8.

To study the proteome landscape in the infected urinary bladder, we infected mice with UPEC and studied the local immune response by a novel spatial imaging technique. To this end, we established the algorithm SPRING (Spatial PRoteome ImagiNG), which employs computational coregistration methods to analyze mass spectrometry datasets and extract spatial and molecular information in biological samples. In order to indicate the differentially regulated molecules in the connective tissue and urothelium after infection, we determined the discriminating peptides by MALDI-MSI. This unsupervised analysis revealed many peptides with significant differential expression in the connective tissue and urothelium (Fig. 1a, b). Next, SPRING was used to perform a discovery-driven pathway analysis on the differentially expressed proteins in the connective tissue and the urothelium. Such network analysis of the Gene Ontology pathway *“Immune system process”* indicated a strong cluster for chemotaxis and migration, mainly including myeloid cells, such as neutrophils and macrophages (Fig. 1c, d; Table S1). Notably, annotations for macrophage chemotaxis and migration were most indicative in the urothelial analysis, suggesting migration of macrophages from the connective tissue into the infected urothelium.

To specifically study the migration of macrophages into the urothelium, the annotations for the gene ontology (GO) terms “macrophage migration” (GO:1905517) and “macrophage activation” (GO:0042116) were linked to protein accession IDs (Table S2). The expression of the proteins of the GO terms were averaged and a combined distribution map was generated (Fig. 1e). SPRING revealed a strong enrichment of the proteins of both GO terms in the connective tissue as well as the urothelium and lumen (Fig. 1e), the entry site for invading UPEC. Analysis of the individual proteins of the GO term “macrophage migration” in the urothelium revealed strong expression of Myo9b (Fig. 1f), a molecule important for chemokine-induced attraction of macrophages.^25^ Moreover, the protein Tim3, which contributes to the elimination of apoptotic bodies,^26^ was strongly expressed in the urothelium (Fig. 1f). These data suggest macrophage-specific alterations within the infected urothelium. Furthermore, Myo9b reached the highest spatial correlation between F4/80 and the proteins of the indicated GO terms (Fig. 1g). These data show the presence of proteins associated with macrophage migration in the infected urothelium.

### Macrophages relocate into the urothelium upon UPEC infection

**Fig. 2:**
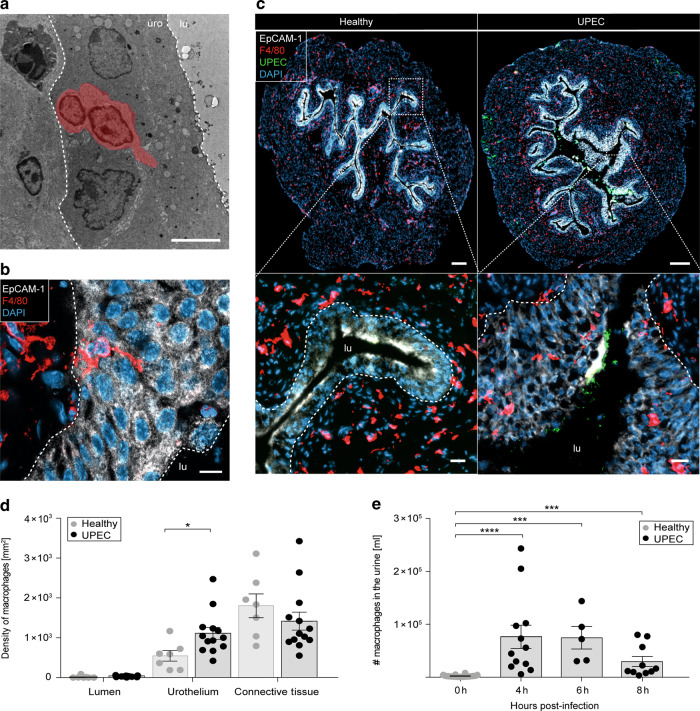
Macrophages accumulate within the infected urothelium. Mice were infected with UPEC and analyzed 1 day post-infection (**a**–**d**) or at the timepoints indicated (**e**). **a** Electron microscopy of bladder tissue sections after UPEC infection. The scale bar indicates 10 µm. The macrophage was pseudo-colored based on its cellular structure and protrusions. **b** Detection of intraurothelial macrophages by confocal microscopy. The white dashed lines separate the urothelium from the connective tissue (left dashed line) and the urothelium from the lumen (right dashed line). The scale bar indicates 10 µm. **c** Immunofluorescence microscopy revealed migration of macrophages towards the infection. The scale bars indicate 200 µm (top images) and 30 µm (bottom images). The white dashed lines represent the border between the urothelium and the connective tissue. **d** The density of F4/80^+^ cells was collected by SCHNELL by microscopy (**c**) in bladder tissue compartments. **e** Longitudinal study of F4/80^+^ macrophages in the urine using flow cytometry (0 h: *n* = 24, 4 h: *n* = 12, 6 h: *n* = 5, 8 h: *n* = 10). *p < 0.05; ***p < 0.001; ****p < 0.0001. Error bars show the mean ± SEM. lu, lumen; uro, urothelium.

To further study the appearance of macrophages within the urothelium during acute bacterial infection of the urinary bladder, tissue sections were analyzed by electron and immunofluorescence microscopy. We found intraurothelial cells with protrusions and a cellular shape reminiscent of macrophages by electron microscopy (Fig. 2a). Immunofluorescence microscopy detected F4/80^+^ macrophages within the EpCAM-1^+^ urothelium (Fig. 2b and S3) and these cells were in close proximity to invading UPEC (Fig. 2c). To establish an automated and non-biased quantification of macrophages, we developed the algorithm SCHNELL (Statistical Computing on Histology Networks Enabling Leukocyte Location). Macrophage location and abundance were assessed by SCHNELL after segmenting the bladder tissue by the urothelial-specific marker EpCAM-1 and using F4/80 and DAPI for macrophage identification. SCHNELL determined a significant number of macrophages that appeared close to the site of infection and an increased density was detected within the urothelium (Fig. 2d). Moreover, macrophages were present in the urine already 4 h after inoculating UPEC into the bladder (Fig. 2e and S4), corroborating the finding of macrophages relocation during acute bacterial infection in the urinary bladder. These findings confirm the SPRING-based results and demonstrate macrophage accumulation at the site of urothelial infection.

### Intraurothelial macrophages phagocytose UPEC and eliminate neutrophils

**Fig. 3:**
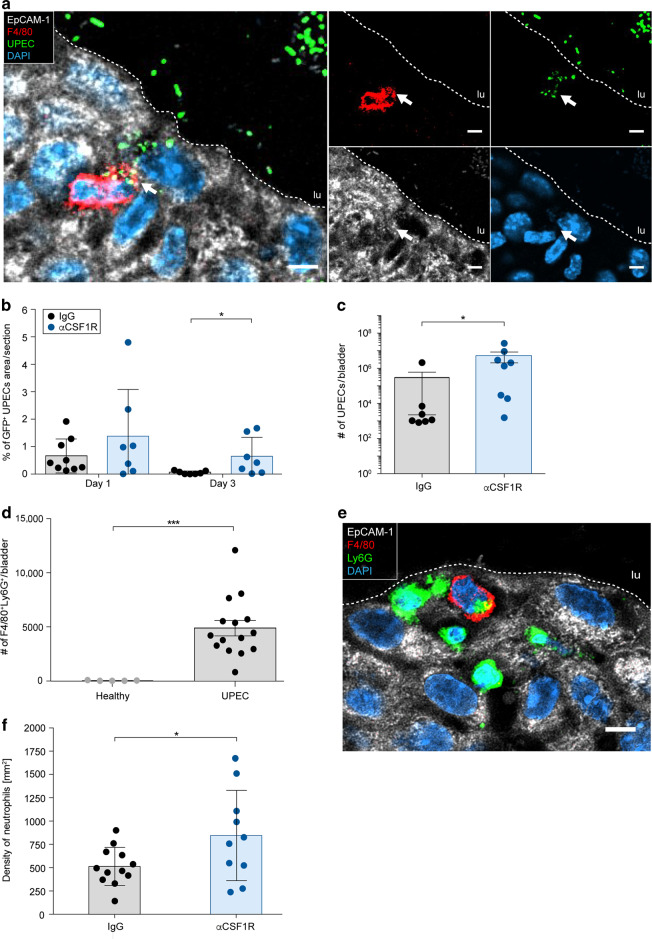
Intraurothelial macrophages phagocytose neutrophils and UPEC. **a** Confocal microscopy demonstrating phagocytosis of GFP^+^ UPEC (green) by F4/80^+^ macrophages (red) in the EpCAM-1^+^ urothelium (white) 1-day post-infection. **b** GFP^+^  UPEC in the urinary bladder determined by fluorescent image analysis (SCHNELL) after macrophage depletion by intraperitoneal application of αCSF1R antibodies. **c** The colony forming units of UPEC were determined in bladder homogenates three days post-infection. **d** Flow cytometry-based quantification of phagocytosis of neutrophils by macrophages (F4/80^+^Ly6G^+^) in bladder digests 1-day post-infection. **e** Confocal microscopy demonstrating phagocytosis of Ly6G^+^ neutrophils (green) by F4/80^+^ macrophages (red) in the EpCAM-1^+^ urothelium (white) 1-day post-infection. **f** Density of neutrophils in the urinary bladder after macrophage depletion by intraperitoneal application of αCSF1R antibodies 1-day post-infection. *p < 0.05, ***p < 0.001. Error bars show the mean ± SEM. lu=lumen. The scale bars in **a**, **d** indicate 5 µm and the white dashed lines distinguish the EpCAM-1^+^ urothelium from the lumen.

Phagocytosis of pathogens is critical to fight off infections and may prevent invasion of UPEC into deeper bladder tissue. Earlier in this study, we have shown that Tim3 was strongly upregulated and its expression correlated with the presence of macrophages (Fig. 1). Thus, we considered phagocytosis of UPEC as an important defense mechanism which is facilitated by relocated macrophages. To study the uptake of bacteria, we inoculated GFP-tagged UPEC into the urinary bladder and imaged intracellular UPEC by confocal microscopy. Macrophages were efficient in phagocytosing UPEC within the urothelium (Fig. 3a). Depletion of macrophages by injection of antibodies against the CSF1 receptor (CSF1R) (Fig. S5A, B) increased the bacterial burden (Fig. 3b, c and S5C). Particularly, the infection strength on day 3 was strongly increased after macrophage depletion by microscopy (Fig. 3b) and bladder homogenates (Fig. 3c). Using flow cytometry and confocal microscopy, we also found Ly6G^+^ neutrophils within intraurothelial macrophages (Fig. 3d, e and S6) and depletion of macrophages increased the number of neutrophils in the infected bladder (Fig. 3f and S7). These data indicate that macrophages are critical to contain the infection and phagocytose neutrophils.

### Blockade of IL-6 reduces macrophage migration and aggravates acute bacterial infection of the urinary bladder

**Fig. 4:**
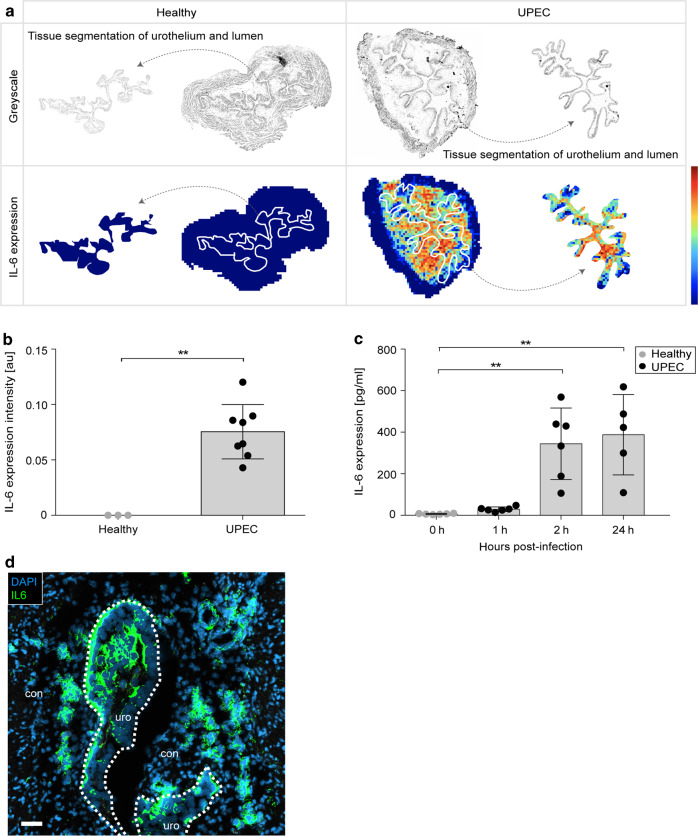
Expression of IL-6 in the urinary bladder tissue compartments upon UPEC infection. **a**, **b** Mice were infected with UPEC and analyzed 1-day post-infection. **a** Representative spatial distribution of IL-6 in healthy (left) and UPEC-infected (right) bladders by MALDI-MSI. The top row shows a greyscale image and the tissue segmentation of the urothelium from the urinary bladder. The bottom row demonstrates the expression of IL-6. The white lines separate the connective tissue from the urothelium and the lumen. The segmented urothelium and lumen are represented as individual images on the far right and far left sides. **b** Within the urothelium, the average intensity of IL-6 per pixel per sample was collected. **c** Longitudinal study on the concentration of IL-6 in bladder homogenates by ELISA (0 h: *n* = 6, 1 h: *n* = 6, 2 h: *n* = 6, 24 h: *n* = 5). **d** Detection of IL-6 on cryosections 3 h after infection by immunofluorescence microscopy. Scale bar indicates 50 µm and dashed lines separate the urothelium from the connective tissue. **p < 0.01. Error bars show the mean ± SEM. con, connective tissue; uro, urothelium.

**Fig. 5:**
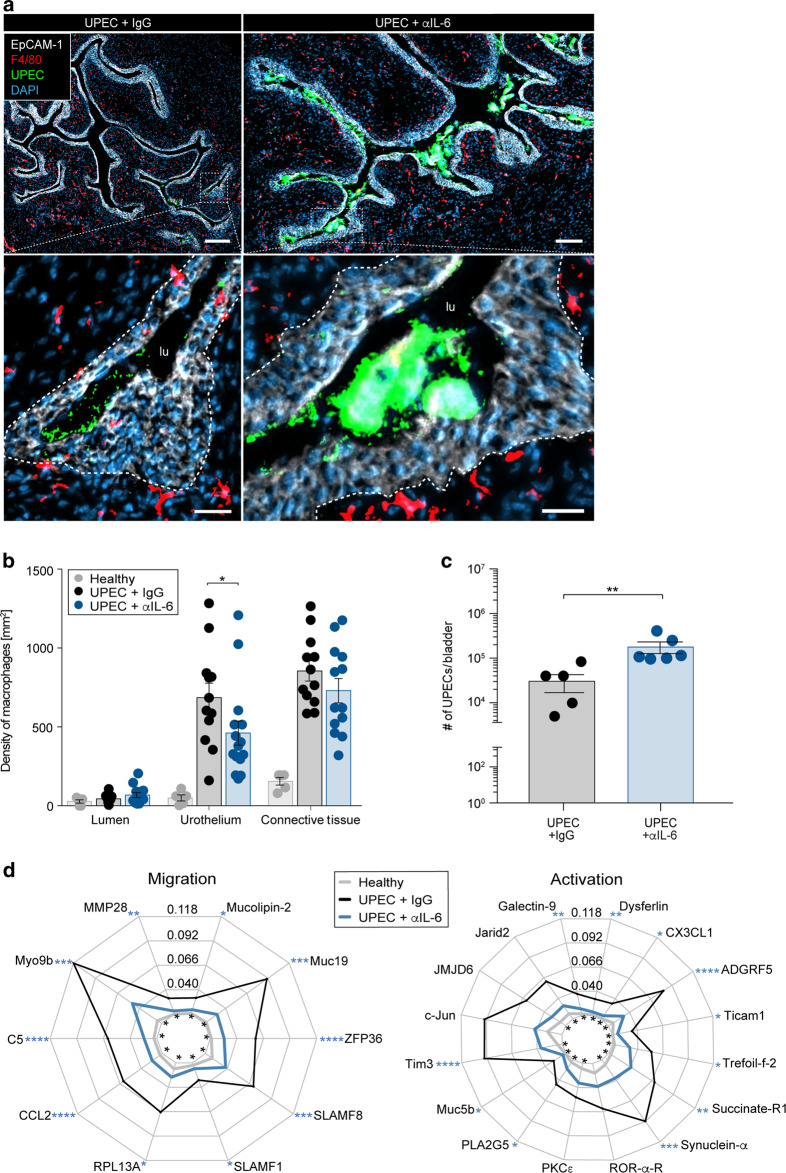
Macrophage relocation and the defense against UPEC depends on IL-6. Mice were infected with GFP-expressing UPEC and analyzed 1-day post-infection. IL-6 expression was topically inhibited by inoculating IL-6 antibodies (αIL-6) into the urinary bladder. Inoculating IgG antibodies served as the control. **a** Bladder tissue sections were stained with DAPI (blue), F4/80 (red) and EpCAM-1 (white) and the images were acquired by a fluorescence microscope. Dashed lines separate the urothelium from the connective tissue. The scale bars in the top row indicate 200 µm, bottom row 30 µm. **b** The density of F4/80^+^ macrophages in the bladder compartments was calculated by SCHNELL on immunofluorescent images. **c** The colony forming units of UPEC were determined in bladder homogenates in the presence and absence of IL-6. **d** Spider plots of the expression intensities [au] of the proteins of the GO terms “macrophage migration” (GO: 1905517) and “macrophage activation” (GO: 0042116) in the urothelium determined by MALDI-MSI. Asterisks inside the circle refer to the statistical analysis between healthy and UPEC infected (* in black), whereas the asterisks outside the spider plot (* in blue) compares UPEC versus αIL-6. *p < 0.05, **p < 0.01. Error bars show the mean ± SEM; lu, lumen.

In order to study the mechanisms that mediate the accumulation of urothelial macrophages, the non-targeted SPRING dataset was analyzed for molecules highly expressed in the urothelium. SPRING revealed strong upregulation of IL-6 (Fig. 4a, b) and the expression of this molecule was also strongly detected in bladder homogenates post-infection (Fig. 4c) and by an antibody-based detection on bladder sections (Fig. 4d). To study the relevance of IL-6 for macrophage localization in the urothelium and bacterial burden, IL-6 antibodies were topically inoculated into the urinary bladder. We found a significant reduction of intraurothelial macrophages (Fig. 5a, b) and an increased bacterial burden by microscopy (Fig. 5a) and in tissue homogenates (Fig. 5c). We also observed an impeded expression of proteins involved in macrophage migration and activation in the urothelium after blocking IL-6 (Fig. 5d and S8). These data demonstrate the critical role of IL-6 during acute bacterial infection in the urinary bladder and identify potential proteins involved in macrophage relocation into the infected urothelium.

### CX_3_CL1 expression depends on IL-6 and mediates macrophage migration into the urothelium

**Fig. 6:**
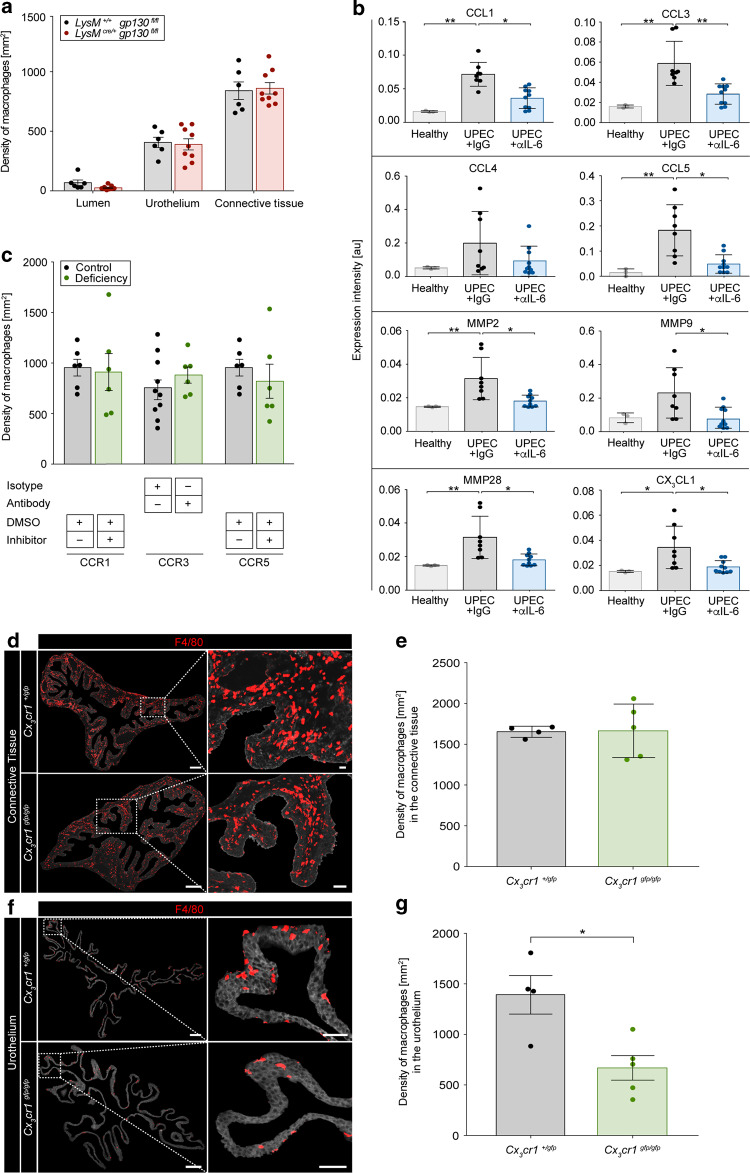
CX_3_CL1 expression depends on IL-6 and mediates macrophage migration into the urothelium. Mice were infected with UPEC and analyzed 1-day post-infection. **a** The density of F4/80^+^ macrophages, in which macrophages were either deficient (*LysM*^*cre/+*^ *gp130*^*fl/fl*^) or competent (*LysM*^*+/+*^ *gp130*^*fl/fl*^) in IL-6 receptor signaling, was calculated by SCHNELL on immunofluorescent images. **b** Detailed expression intensities [au] of the chemokines indicated within the urothelium by MALDI-MSI. **c** The urothelial density of F4/80^+^ macrophages was determined by immunofluorescence microscopy after topical treatment with CCR1 (control: DMSO), CCR3 (control: IgG2b) and CCR5 (control: DMSO) inhibitors. **d**–**g** Bladder tissue sections from fractalkine receptor competent (*Cx*_*3*_*cr1*^*+/gfp*^) and -deficient (*Cx*_*3*_*cr1*^*gfp/gfp*^) mice were stained with F4/80 (red) and acquired by fluorescence microscopy. The connective tissue is shown on the top panel (**d**, **e**), and urothelium on the bottom panel (**f**, **g**). The scale bars in the first column indicate 200 µm, second column 50 µm. **e**, **g** The density of F4/80^+^ macrophages was calculated by SCHNELL on the immunofluorescent images (Representative images shown in **d**, **f**). *p < 0.05, **p < 0.01. Data are means ± SEM.

The molecule IL-6 induces classical IL-6 signaling after binding to the IL-6 receptor and recruitment of gp130. Alternatively, IL-6 bound to the cleaved IL-6 receptor activates trans IL-6 signaling via direct binding of the fusion protein to gp130. Macrophages in *LysM*^*cre/+*^ *gp130*^*fl/fl*^ animals were still able to migrate into the urothelium (Fig. 6a). This indicates that IL-6 acts on urothelial cells to facilitate relocation of macrophages. As presented in Fig. 1d, Myo9b showed the strongest correlation with macrophages in the infected urothelium, indicating chemokine-mediated migration of macrophages.^25^ Among others, CCL1, CCL3, CCL5, and CX_3_CL1 were significantly altered in the absence of IL-6 (Fig. 6b). However, inhibition of the corresponding receptors, namely CCR1, CCR3 and CCR5, did not reduce the number of macrophages (Fig. 6c). However, targeting CX_3_CR1 did not impede the number of macrophages in the connective tissue (Fig. 6d, e), but markedly reduced their abundance in the urothelium (Fig. 6f, g).

## Discussion

Macrophages are found throughout the body, where they have crucial roles in tissue development, homeostasis and remodeling, as well as maintaining the barrier function of epithelial interfaces. Using matrix-assisted laser desorption/ionization mass spectrometry imaging (MALDI-MSI) and liquid chromatography mass spectrometry (LC-MS/MS), we now provide evidence that macrophages in the urinary bladder relocate from the connective tissue into the UPEC-infected urothelium in an IL-6-induced and CX_3_CL1-dependent manner.

The bladder has an almost impenetrable urothelium, which protects the host tissue from substances that accumulate in the urine and prevents the invasion of microorganisms.^27,28^ Such physical barrier establishes the first line of defense and responds to potential infections by producing inflammatory molecules and recruiting leukocytes. In the intestine, numerous macrophages were also observed in the lamina propria underneath the epithelium^29,30^ and the chemokine receptor CX_3_CR1 was found to be critical for the formation of transepithelial dendrites (TED) to access intestinal antigens.^31^ An earlier study also detected macrophage protrusions within the urothelium of the urinary bladder under homeostasis.^32^ This finding suggests that signals derived from the urothelium or the lumen may shape immunological responses by macrophages. Recent studies suggested that in addition to TEDs, transepithelial migration of intestinal macrophages prevents *Salmonella* from traversing the epithelium.^33^ The mechanisms for this migratory step however remained unknown. Our study demonstrates that macrophages migrate into the multilayered urothelium of the urinary bladder during UPEC infection. In this study, we provide a novel immunological mechanism on how the multilayered urothelium actively stimulates relocation of macrophages upon infection. Such active migration is facilitated by chemokines and proteinases and among those, IL-6 and CX_3_CL1 were most critical for the entry of macrophages into the urothelium.

Macrophages in epithelial interfaces are constantly exposed to environmental cues from the outside world.^34^ Upon infections, large numbers of leukocytes are recruited and our data demonstrate that neutrophils are eliminated by relocated macrophages within the urothelium. The elimination of apoptotic cells by efferocytosis is a physiological and vital mechanism that avoids overshooting tissue inflammation while allowing the development of antibacterial immune responses.^35^ Previously, the transmembrane protein Tim3 has been shown to capture and eliminate apoptotic bodies after phosphatidylserine union.^26^ Our data indicate a strong and colocalized expression of Tim3 with urothelial macrophages, suggesting that Tim3-expressing macrophages may eliminate neutrophils.

Chemotaxis is defined as directional migration of leukocytes towards a soluble chemical gradient. Migration of macrophages in complex and diverse 3D tissue environments has not extensively been studied. Previously, two principal modes of movement, amoeboid and mesenchymal migration, have been described in tissues, but only the latter depends on proteases.^36^ Phenotypical characterization of macrophages in the urothelium by fluorescence and electron microscopy showed long protrusions with potential actin-rich podosomes, suggesting that these cells rather employ a protease-dependent mesenchymal migration mode in this complex and collagen-rich architecture of the urothelium. Accordingly, reduced migration of macrophages in the absence of IL-6 were correlated with impeded expression of MMP2, 9 and 28. Previously, soluble biomarkers involved in myeloid cell development and chemotaxis could be predictive of acute bacterial infection recurrence.^37^ Our algorithm SPRING also colocalized macrophages with Myo9b upon infection, an important molecule for chemokine-induced attraction of macrophages.^25^ Previously, CX_3_CL1 was proven to be critical in intestinal inflammatory disorders and for sampling of bacteria from the intestinal lumen.^31^ Moreover, deficiency in CX_3_CR1 increased translocation of commensal bacteria to the gut draining lymph node.^38^ However, the signals that regulate the expression of the ligand, CX_3_CL1, are poorly understood. Analysis of the CX_3_CL1 promoter indicated the presence of DNA binding *cis*-elements for various inflammatory modulators, such as nuclear factor-κB (NF-κB), signal transducer and activator of transcription (STAT)1/STAT3, and activator protein-1 (AP-1) within their promotor regions.^39,40^ Moreover, transient overexpression of STAT1/STAT3 increased CX_3_CL1 promoter activity and chromatin immunoprecipitation assays revealed the existence of physical interactions of STAT1/STAT3 with elements of the CX_3_CL1 promoter.^39^ Accordingly, Interferon has been indicated to upregulate CX_3_CL1 expression through STAT.^41,42^ Our data adds an important inflammatory molecule to the list of CX_3_CL1 inducers and suggests that IL-6-dependent STAT signaling has the capacity to bind to the promotor region of CX_3_CL1.

Recently, uptake of UPEC in an ATG16L1-dependent manner^13^ and iron retention by macrophages in the urinary bladder have been observed.^11,12^ Our study demonstrates that relocated macrophages in the urothelium phagocytose UPEC to reduce infection. Hence, this process of phagocytosis of UPEC is an important antimicrobial mechanism during acute infections of the urinary bladder. Neutrophils have also been shown to phagocytose and eliminate UPEC.^6^ Moreover, we detected phagocytosis of neutrophils by macrophages in the urothelium. However, we did not observe macrophages positive for both marker, Ly6G from neutrophils and GFP from UPEC. A possible explanation is that the UPEC-specific GFP signal may be lost after phagocytosis.

The mechanistic investigation of macrophage relocation was facilitated by the novel coregistration algorithm SPRING. Using SPRING, we were able to generate expression landscapes for proteins of the GO terms “macrophage migration” and “macrophage activation”. Spatial coregistration analysis of the prototypical macrophage marker F4/80 with the proteins of the GO terms indicated a strong increase in the molecules Myo9b and Tim3. Thus, both molecules critically shape macrophage behavior during acute bacterial infection in the urinary bladder and our algorithm SPRING was able to identify these changes. Notably, the resolution limit of MALDI-MSI (50 µm^2^) might explain the low correlation factors in the linear regression analysis between F4/80 and the proteins of the GO terms. Accordingly, the expression intensity collected in a single pixel might be affected by many different cell types, indicating that technical advancement and increased resolution will be required to achieve cellular resolution. However, segmented and unbiased analysis of larger specimens, such as the urothelium, solved the cellular resolution limitation and SPRING was able to identify the proteome landscape that facilitated the relocation of macrophages into the urothelium. Importantly, SPRING exploited the label-free MALDI-MSI dataset in both an untargeted and targeted proteomics approach, indicating that the spatial distribution of any molecule and cellular marker of interest could be mapped and colocalized by SPRING.

One of the molecules highly expressed in the urothelium upon UPEC infection was IL-6. Pathogen recognition by urothelial cells has been suggested to stimulate IL-6 secretion^43^ with key roles in limiting urothelial invasion and ascending infection.^44^ Analysis by SPRING revealed strong upregulation of this molecule within the infected urothelium and topical inhibition by local intraurethral administration of antibodies against IL-6 aggravated the infection. Interestingly, the expression of IL-6 strongly correlates with bacterial burden in humans^45,46^ and local production of IL-6 by urothelial cells was recently suggested.^47^ Importantly, application of the JAK-STAT inhibitor Ruxolitinib, which also inhibits IL-6 signaling, predisposed patients for acute bacterial infection in the urinary bladder,^48^ suggesting a critical role of IL-6-signaling for infection control. Analysis of the microenvironment in the urothelium by SPRING and immunofluorescence microscopy indicated that indeed urothelial cells produced this pleiotropic molecule and topical inhibition of IL-6 within the urinary bladder reduced the chemokines CCL1, CCL3, CCL4, CCL5, and CX_3_CL1. Finally, specific inhibition of the corresponding chemokine receptors identified CX_3_CR1 as most critical for macrophage relocation into the urothelium, indicating that IL-6 shapes the local urothelial microenvironment for CX_3_CL1-dependent macrophage relocation.

In conclusion, our findings support a model in which macrophages reduce bacterial burden by phagocytosis of UPEC, thereby executing a tissue-protective role. The migratory dynamics of macrophages represent a core function evolved to respond to bacterial infection with UPEC, which may also reduce tissue damage by phagocytosis of apoptotic neutrophils. We conclude that our correlative algorithm SPRING provided novel insights during infections with UPEC and unraveled the mechanism of macrophage relocation. SPRING identified the critical crosstalk between urothelial cells and macrophages through IL-6 and CX_3_CL1 indicating that this novel algorithm holds the potential to reveal important cellular and proteomic landscapes in an untargeted approach contained in any dataset. Finally, the IL-6-induced and CX_3_CL1-dependent crosstalk of urothelial cells and macrophages facilitated the relocation of macrophages into the infected urothelium, probably provoking a rather resolving and regenerative environment and reducing bacterial burden caused by UPEC.

## Materials and Methods

*Animal studies*. Female C57BL/6 mice were used throughout the experiments. Animals were purchased from Jackson Laboratories or bred and maintained under specific-pathogen-free conditions in the central animal facility at the University Hospital Essen. The following mouse strains were used for the study:

- C57BL/6J
- F2 crossbreed: *LysM*^*cre/+*^; B6.129P2-*Lyz2*^*tm1(cre)Ifo*^/J; Il6st^tm1.1Wme^
- *Cx*_*3*_*cr1*^*gfp/gfp*^; B6.129P2(Cg)-*Cx*_*3*_*cr1*^*tm1Litt*^/J

Animal experiments were approved by the local animal review boards (Bezirksregierung Köln, Landesamt für Natur, Umwelt und Verbraucherschutz NRW in Recklinghausen, Germany).

*Acute bacterial infection in the urinary bladder model.* Uropathogenic *E. coli* (UPEC) strain 536 (O6:K15:H31) and 536^gfp^^49^ were cultured for 3 h at 37 °C in LB medium. Bacteria were harvested via centrifugation at 1500 × *g* for 20 min and suspended in 1 ml of PBS. Female mice were anesthetized with a 1:1 mixture of 2% Xylazine and 10% Ketamine. The animals were then infected via transurethral inoculation of 5 × 10^8^ UPEC in 0.05 ml PBS using a soft polyethylene catheter.

*Blocking experiments and macrophage depletion*. Blocking experiments were performed by transurethral injection of 1.5 µg/g mouse weight of the indicated antibodies and inhibitors (key resource table) into the bladder lumen 1 h post infection. Anti-CSF1R (αCSF1R) antibody was used to deplete bladder macrophages. Animals received two intraperitoneal injections of αCSF1R antibody or isotype control (20 μg/g mouse weight on day 1 and 10 μg/g mouse weight on day 2 before infection).

*Isolation of leukocytes from the urinary bladder*. Bladders were sliced into small pieces using a scalpel and then digested for 45 min at 37 °C with 0.5 mg/ml collagenase and 100 mg/ml DNAse I in RPMI 1640 medium supplemented with 10% heat-inactivated FCS, 20 mM HEPES, 1 mM L-glutamine and antibiotics. Single-cell suspensions were filtered through a 100 mm nylon mesh and analyzed by flow cytometry.

*Bacterial burden by colony* *forming units.* Bacterial burden was determined by plating different dilutions of homogenate of enzymatically digested urinary bladders on CPS agar plates overnight at 37 °C. Colony forming units (CFU) were counted and the number of CFU were calculated.

*Detection of IL-6 by ELISA.* Urinary bladders were mechanically homogenized in the presence of proteinase inhibitors and centrifuged at 13,000 rpm for 10 min at 4 °C. The protein levels of IL-6 in the supernatant were determined by using ELISA according to the manufacturer’s protocols.

*Flow cytometry.* Single-cell suspensions were washed with PBS containing 0.1% BSA and 0.1% NaN_3_, and Fc-receptors were blocked with human immune globulin. Titrated amounts of the fluorochrome-labeled antibodies were used for staining. Cells were measured using an LSR Fortessa (BD Biosciences) and analyzed with the FlowJo software 10. Absolute cell numbers were calculated by adding a fixed number of APC-labeled microbeads (BD Biosciences) to each sample.

*Electron microscopy.* Bladder tissue sections were fixed with 3% glutaraldehyde in 0.1 M cacodylate buffer [pH 7.4] followed by 2% osmium tetroxide. Bladder tissue sections were embedded in Epon 812 embedding resin, and 40–50 nm thin sections were cut with an LKB ultramicrotome UM IV (Leica) and analyzed using a CM10 electron microscope (Philips).

*Immunofluorescence microscopy.* Bladder tissues sections were fixed overnight in PLP buffer [pH 7.4, 0.05 M phosphate buffer containing 0.1 M L-lysine, 2 mg/ml sodium periodate, and paraformaldehyde with a final w/v concentration of 4%]. Subsequently, bladder tissue sections were equilibrated in 30% sucrose for 24 h and then frozen in Tissue-Tek OCT. Bladder tissue sectioning was performed at −20 °C by using a cryostat. Sections with a thickness of 10 µm were rehydrated using PBS containing 0.05% Triton X-100, blocked for 1 h with PBS containing 1% BSA and 0.05% Triton X-100, and 2–3 sections per bladder were imaged on a Zeiss AxioObserver.Z1 or Leica SP8 gSTED Super-Resolution confocal and FLIM. For IL-6 immunostaining, animals were sacrificed 3 h post infection and explanted bladders were pre-incubated with 1 µg/ml Golgi-Plug in RPMI [10% FCS, 1% L-glutamine, 1% Penicillin/Streptomycin) for 4 h at 37 °C, snap-frozen in Tissue-Tek OCT and processed for cutting as described above.

### Key resource table

**Table:** 

Reagent or resource	Source	Identifier
*Antibodies*		
CCR3	BioXCell	Cat #BE0316
CSF1R	BioXCell	Cat #BE0213
CD45	BioLegend	Cat #103154
EpCAM-1	BioLegend	Cat #118204
F4/80	eBioscience	Cat #17-4801-80
IgG1, isotype control for IL-6	eBioscience	Cat #16-4301-85
IgG2a, isotype control for CSF1R	BioXCell	Cat #BE0089
IgG2b, isotype control for CCR3	R&D Systems	Cat #MAB0061
IL-6, inhibition experiments	eBioscience	Cat #16-7061-85
IL-6, immunofluorescence microscopy	BD Biosciences	Cat #561367
Ly6G	BioLegend	Cat #127607
*Reagents*		
2-Iodacetamide	Merck	Cat #8.047.440.025
A1B1 hydrochloride (CCR1)	Axon Medchem	Cat #Axon1179
Acetonitril	Biosolve	Cat #0001207802BS
Ammonium hydrogen carbonate	PanReac AppliChem	Cat #A3583,0500
Bovine serum albumin	GE Healthcare	Cat #K45-001
CHCA matrix	Bruker	Cat #8201344
ChromIDTM CPS® Elite agar plates	Biomérieux	Cat # 416172
Collagenase	Sigma-Aldrich	Cat #C2674
DAPI	Life Technologies	Cat #D1306
Dithiothreitol	PanReac AppliChem	Cat #A1101,0025
DMSO, control for CCR1 and CCR5	PanReac AppliChem	Cat #A3672,0250
DNAse I	Sigma-Aldrich	Cat #D4513-1VL
Epon 812	Serva	Cat #21045.01
Fetal calf serum	Biochrom	Cat #S0615
Golgi-Plug	BD	Cat #51-2301KZ
HEPES	Sigma-Aldrich	Cat #H4034
IL-6 Quantikine ELISA Kit	R&D Systems	Cat #M6000B
LB medium	Carl Roth GmbH	Cat #X64.
L-glutamine	Sigma-Aldrich	Cat #G7513
L-lysine	Sigma-Aldrich	Cat #L5626
Maraviroc (CCR5)	SelleckChem	Cat #S2003
Paraformaldehyde	Sigma-Aldrich	Cat #P6148
PBS	Life Technologies	Cat #18912-014
Phosphate buffer [NaH_2_PO_4_ + Na_2_HPO_4_]	Sigma-Aldrich	Cat #71500
	Carl Roth GmbH	Cat #P030.1
Proteinase inhibitor mix	Roche Diagnostics	Cat #4963159001
RapiGest SF surfactant	Waters	Cat #186001861
RPMI 1640	Life Technologies	Cat #42401-42
Sodium periodate (NaIO_4_)	Carl Roth GmbH	Cat #2603.1
Sucrose	Carl Roth GmbH	Cat #9097.1
Tissue-Tek OCT	Sakura	Cat #4583
Trifluoroacetic acid	VWR	Cat #85.049.001
Triton X-100	Carl Roth GmbH	Cat #3051.4
*Experimental models: mouse strains*		
C57BL/6J	Jackson Laboratories	Stock #000664
*Cx*_*3*_*cr1*^*gfp/gfp*^; B6.129P2(Cg)-*Cx*_*3*_*cr1*^*tm1Litt*^/J	Jackson Laboratories	Stock #005582
Il6st^tm1.1Wme^	Betz et al.^50^	10.1084/jem.188.10.1955
*LysM*^*cre/+*^; B6.129P2-*Lyz2*^*tm1(cre)Ifo*^/J	Jackson Laboratories	Stock #004781
Uropathogenic *E. coli* (UPEC) strain 536 (O6:K15:H31)	Berger et al.^51^	Not available
Uropathogenic *E. coli* (UPEC) strain 536^gfp^ (O6:K15:H31)	Engel et al.^49^	10.1128/IAI.00881-06
*Software*		
Adobe Illustrator CC 2018–2019	Adobe	RRID:SCR_010279
Cytoscape 3.7.2 with ClueGO App	Cytoscape	RRID:SCR_003032
Fiji	ImageJ	RRID:SCR_003070
FlowJo 10	FlowJo	RRID:SCR_008520
GraphPad Prism, version 7	GraphPad Software	RRID:SCR_002798
Imaris, versions 8.6–9.3	Bitplane	RRID:SCR_007370
KNIME (Konstanz Information miner)	www.knime.com	RRID:SCR_006164
R Project for Statistical Computing, version 3.5.1	https://cran.r-project.org/	RRID:SCR_001905
SCiLS Lab 3D, 2017a	SCiLS	RRID:SCR_014426
ZEN Digital Imaging for Light Microscopy, ZEN 2012	Zeiss	RRID:SCR_013672

*Analysis of microscopy images by SCHNELL (Statistical Computing of Histology Networks Enabling Leukocyte Location).* An intensity threshold was used to generate masks for each fluorescent channel and the binary information for cellular and nuclear signals was coregistered. Automated analysis of cell densities was performed by a Java based algorithm SCHNELL. Using ImageJ, overlapping mask regions were employed to identify cells, which were marked with a point at the center of the DAPI^+^ cell nucleus. The bladder tissue was segmented into lumen, urothelium and connective tissue by employing the EpCAM-1 signal and cell densities were calculated.

*Matrix-assisted laser desorption/ionization imaging (MALDI-MSI).* Bladders were perfused transcardially with a total volume of 20 ml PLP buffer [pH 7.4, 0.05 M phosphate buffer containing 0.1 M L-lysine, 2 mg/ml sodium periodate, and paraformaldehyde with a final w/v concentration of 4%] at a perfusion rate of 4 ml per minute. Perfused bladder tissue was fixed in PLP buffer overnight. Fixed bladder tissue was equilibrated in 30% sucrose for 24 h and 5 µm sections were cut at −20 °C using a cryostat. The slices were mounted on indium tin oxide (ito) slides. Tissue sections were washed with ethanol (70% and 95%) for 2 min. The following steps of on-section tryptic digestion and application of CHCA matrix were performed as described previously.^52^ Measurements were performed using the UltrafleXtreme mass spectrometer from Bruker. The parameters were as follows: reflective positive ion mode, mass range: 700–4000 *m/z*, spatial resolution: 50 µm^2^, laser beam size: medium, shots per position: 300–500, sampling rate: 2.5 GS/s. For external instrument calibration the Bruker peptide calibration standard was spotted next to the tissue section. The SCiLS Lab software was used for data and imaging analysis of the MSI measurements. All data underwent a total ion count (TIC) normalization and a baseline removal.

*Liquid chromatography-mass spectrometry (LC-MS/MS).* Bladder tissue sections were scraped off the glass slides and lysed by addition of 20 µl ammonium bicarbonate (50 mM), containing 0.1% RapiGestSF surfactant per sample. Disulfide bonds were reduced with 5 mM dithiothreitol at 60 °C for 30 min and alkylated with 15 mM iodoacetamide for 30 min at ambient temperature in the dark. Lysed proteins were digested with trypsin overnight at 37 °C using 0.1 μg trypsin per sample. For acidification, trifluoroacetic acid (TFA) was added (0.5%, 30 min, 37 °C) and the samples were centrifuged (10 min, 16,000 × *g*) for removal of precipitated RapiGest. The supernatants were collected, dried in a vacuum centrifuge, and dissolved in 50 mM ammonium bicarbonate. For peptide purification, Sera-Mag beads were equilibrated with water and peptides were added to the beads (40 µg beads per sample, hydrophilic and hydrophobic beads mixed 1:1), before acetonitrile (ACN) was added to a final fraction of 95%. Peptides were allowed to bind for 10 min at room temperature before the beads were immobilized using a magnetic rack. Beads were washed with ACN two times, dried and 20 µl 0.1% TFA per sample were added. The samples were sonicated on ice for 5 min and the eluted peptides were separated from the beads using a magnetic rack. The LC-MS/MS measurement was performed using an Ultimate 3000 RSLCnano system coupled online to an Orbitrap Elite mass spectrometer (both Thermo Scientific). Peptides dissolved in 0.1% TFA were pre-concentrated on a C18 trap column (Acclaim PepMap 100; 100 μm × 2 cm, 5 μm, 100 Å; Thermo Fisher Scientific) within 7 min at a flow rate of 30 μl/min with 0.1% TFA. Subsequently, the peptides were separated on an analytical column (in-house packed C18 analytical column, ReproSil®-Pur (Dr. Maisch HPLC GmbH, Ammerbuch, Germany), 75 μm × 40 cm, 1.9 μm, 120 Å) by a gradient from 5 to 40% solvent B over 98 min (solvent A: 0.1% FA, solvent B: 0.1% FA, 84% ACN; flow rate 300 nl/min; column oven temperature 65 °C). The instrument was operated in a data-dependent mode. Full-scan mass spectra in the Orbitrap analyzer were acquired in profile mode at a resolution of 60,000 at 400 m/z and within a mass range of 350–2000 *m/z*. MS/MS spectra were acquired in data-dependent mode at a resolution of 5400. For MS/MS measurements, the 20 most abundant peptide ions were fragmented by collision-induced dissociation (CID, NCE of 35) and measured for tandem mass spectra in the linear ion trap.

*Spatial proteome imaging (SPRING): an algorithm combining computational and coregistration methods to analyze mass spectrometry datasets.* SPRING combines LC-MS/MS and MALDI-MSI datasets to generate tissue proteome landscapes. In detail, matrix-assisted laser desorption ionization/mass spectrometry imaging (MALDI-MSI) collected a mass spectrum [*m/z*] at each pixel on the tissue sections. In order to determine the identity of a specific *m/z* value, liquid chromatography mass spectrometry (LC-MS/MS) was performed and the proteins were detected based on mass matching to databases by the modular pipelining concept KNIME and the Protein Inference Algorithm (PIA).^24^ The LC-MS/MS data were matched against a *Mus musculus* FASTA database export of UniProt^53^ (release 2018_06, 73045 entries) together with the common Repository of Adventitious Proteins (cRAP, 115 entries). The expression pattern of all peptides linked to the same protein were averaged. For each target protein, one shuffled decoy entry was added to estimate the false discovery rate. The spectrum identification was performed using X!Tandem,^54^ the FDR was estimated and filtered on a 1% level using PIA.^24^ PIA provided information on the *m/z* value of the peptides which included the corresponding protein. For MALDI-MSI, the coordinates and expression intensities of all peptides [*m/z*] per pixel were extracted by SCiLS Lab after background subtraction. These values were correlated to the corresponding peptides [*m/z*] in the LC-MS/MS dataset by SPRING. For proteins linked to more than one peptide, the distribution per pixel was averaged in order to extract one spatial distribution map per protein and sample, as well as spatial distribution maps.

*Pathway enrichment analysis*. We used the software Cytoscape, coupled with the App ClueGO,^55^ to perform the enrichment analysis on the proteins that were significantly upregulated in the urothelium and connective tissue upon infection. The peptides that were used to annotate the proteins for the enrichment analysis had at a fold-change >2 and a *p*-value <0.01. The Gene Ontology databases were updated on the 21.11.2019. The parameters were as follows: any node shown in the network has found at least three genes that belong to this specific pathway, the network specificity was set to medium and the “Immune System process” pathway was used.

### Statistical tests

The volcano plots were generated using a Student *t*-test, and the *p*-values are displayed as –log_10_ in the *y*-axis. Fold-changes are displayed as log_2_ in the *x*-axis. Results are presented as means ± SEM and *p*-values are depicted in the figures. Unpaired Mann–Whitney (two-tailed) or Kruskal–Wallis tests were performed to compare two or more groups. Where applicable, multiple comparison post-hoc corrections were applied (Dunn´s for multiple groups). Paired data from the longitudinal experiments were analyzed using Friedman´s rank test.

## Supplementary information

Supplementary Information
