# Supplementary material for: Spatial proteomics revealed a CX3CL1-dependent crosstalk between the urothelium and relocated macrophages through IL-6 during an acute bacterial infection in the urinary bladder
Source: Mucosal Immunol. 2020 Feb 28;13(4):702–14. doi: 10.1038/s41385-020-0269-7 (PMC7312419; doi:10.1038/s41385-020-0269-7)
Supplement: Supplementary file 1 — Supplementary Information [file 41385_2020_269_MOESM1_ESM.docx]

**SUPPLEMENTAL INFORMATION**

**Table S1. List of the Gene Ontology Terms found in the Enrichment analysis. Related to Figure 1C, D.**

List of the GO terms of the annotated and colored clusters shown Figure 1C and D. The macrophage-related terms are displayed in bold.

**UROTHELIUM**

**Chemotaxis and migration**

- Dendritic cell chemotaxis GO:0002407
- Granulocyte chemotaxis GO:0071621
- Granulocyte migration GO:0097530
- Lymphocyte chemotaxis GO:0048247
- Leukocyte tethering or rolling GO:0050901
- **Macrophage chemotaxis** **GO:0048246**
- **Macrophage migration** **GO:1905517**
- Monocyte chemotaxis GO:0002548
- Negative regulation of leukocyte chemotaxis GO:0002689
- Negative regulation of leukocyte migration GO:0002686
- Negative regulation of lymphocyte migration GO:2000402
- Neutrophil chemotaxis GO:0030593
- Neutrophil extravasation GO:0072672
- Neutrophil migration GO:1990266
- Positive regulation of cellular extravasation GO:0002693
- Positive regulation of leukocyte chemotaxis GO:0002690
- Positive regulation of leukocyte migration GO:0002687
- Positive regulation of lymphocyte migration GO:2000403
- **Positive regulation of macrophage chemotaxis** **GO:0010759**
- **Positive regulation of macrophage migration** **GO:1905523**
- Positive regulation of monocyte chemotaxis GO:0090026
- Positive regulation of mononuclear cell migration GO:0071677
- Positive regulation of neutrophil migration GO:1902624
- Positive regulation of T cell migration GO:2000406
- Regulation of cellular extravasation GO:0002691
- Regulation of granulocyte chemotaxis GO:0071622
- Regulation of leukocyte chemotaxis GO:0002688
- Regulation of lymphocyte chemotaxis GO:1901623
- Regulation of lymphocyte migration GO:2000401
- **Regulation of macrophage chemotaxis** **GO:0010758**
- **Regulation of macrophage migration**  **GO:1905521**
- Regulation of monocyte chemotaxis GO:0090025
- Regulation of mononuclear cell migration GO:0071675
- Regulation of neutrophil migration GO:1902622
- Regulation of T cell migration GO:2000404
- T cell chemotaxis GO:0010818
- T cell extravasation GO:0072683
- T cell migration GO:0072678

**Leukocyte activation**

- Granulocyte differentiation GO:0030851
- **Macrophage activation** **GO:0042116**
- **Macrophage differentiation** **GO:0030225**
- Microglial cell activation GO:0001774
- Mononuclear cell differentiation GO:1903131
- Monocyte differentiation GO:0030224
- **Positive regulation of macrophage activation** **GO:0043032**
- **Regulation of macrophage activation** **GO:0043030**
- Regulation of microglial cell activation GO:1903978

**Mucosal antimicrobial response**

- Antimicrobial humoral immune response mediated by antimicrobial peptide GO:0061844
- Antibacterial humoral response GO:0019731
- Antimicrobial humoral response GO:0019730
- Mucosal immune response GO:0002385

**Cytokine production**

- CD4-positive, alpha-beta T cell cytokine production GO:0035743
- Cytokine secretion involved in immune response GO:0002374
- Dendritic cell cytokine production GO:0002371
- Myeloid leukocyte cytokine production GO:0061082
- Positive regulation of cytokine production involved in immune response GO:0002720
- Positive regulation of cytokine secretion involved in immune response GO:0002741
- Positive regulation of myeloid leukocyte cytokine production GO:0061081

involved in immune response

- Positive regulation of production of molecular mediator of immune response GO:0002702
- Regulation of cytokine production involved in immune response GO:0002718
- Regulation of cytokine secretion involved in immune response GO:0002739
- Regulation of dendritic cell cytokine production GO:0002730
- Regulation of production of molecular mediator of immune response GO:0002700
- Regulation of T cell cytokine production GO:0002724
- T cell cytokine production GO:0002369

**TLR and pattern recognition**

- Activation of innate immune response GO:0002218
- Innate immune response-activating signal transduction GO:0002758
- Negative regulation of toll-like receptor signaling pathway GO:0034122
- Pattern recognition receptor signaling pathway GO:0002221
- Positive regulation of innate immune response GO:0045089
- Positive regulation of toll-like receptor signaling pathway GO:0034123
- Regulation of innate immune response GO:0045088
- Regulation of toll-like receptor signaling pathway GO:0034121
- Toll-like receptor 2 signaling pathway GO:0034134
- Toll-like receptor 4 signaling pathway GO:0034142
- Toll-like receptor signaling pathway GO:0002224

**Fc receptor signaling**

- Fc receptor mediated stimulatory signaling pathway GO:0002431
- Fc receptor signaling pathway GO:0038093
- Fc-gamma receptor signaling pathway GO:0038094
- Fc-gamma receptor signaling pathway involved in phagocytosis GO:0038096
- Immune response-regulating cell surface receptor signaling GO:0002433

pathway involved in phagocytosis

**Complement**

- Complement activation GO:0006956
- Complement activation, alternative pathway GO:0006957
- Complement activation, classical pathway GO:0006958
- Regulation of complement activation GO:0030449

**NK cells**

- Natural killer cell activation GO:0030101
- Natural killer cell activation involved in immune response GO:0002323
- Natural killer cell differentiation GO:0001779
- Natural killer cell mediated cytotoxicity GO:0042267
- Natural killer cell mediated immunity GO:0002228
- Positive regulation of natural killer cell activation GO:0032816
- Positive regulation of natural killer cell mediated cytotoxicity GO:0045954
- Positive regulation of natural killer cell mediated immunity GO:0002717
- Regulation of natural killer cell activation GO:0032814
- Regulation of natural killer cell mediated cytotoxicity GO:0042269
- Regulation of natural killer cell mediated immunity GO:0002715

**CONNECTIVE TISSUE**

**Chemotaxis and migration**

- Granulocyte chemotaxis GO:0071621
- Granulocyte migration GO:0097530
- Lymphocyte chemotaxis GO:0048247
- **Macrophage migration** **GO:1905517**
- Neutrophil chemotaxis GO:0030593
- Neutrophil migration GO:1990266
- Positive regulation of granulocyte chemotaxis GO:0071624
- Positive regulation of leukocyte chemotaxis GO:0002690
- Positive regulation of leukocyte migration GO:0002687
- Positive regulation of lymphocyte migration GO:2000403
- Positive regulation of neutrophil chemotaxis GO:0090023
- Positive regulation of neutrophil migration GO:1902624
- Positive regulation of T cell migration GO:2000406
- Regulation of granulocyte chemotaxis GO:0071622
- Regulation of leukocyte chemotaxis GO:0002688
- Regulation of lymphocyte migration GO:2000401
- **Regulation of macrophage migration** **GO:1905521**
- Regulation of neutrophil chemotaxis GO:0090022
- Regulation of neutrophil migration GO:1902622
- Regulation of T cell migration GO:2000404
- T cell chemotaxis GO:0010818
- T cell migration GO:0072678

**Leukocyte activation**

- Granulocyte activation GO:0036230
- Leukocyte activation involved in immune response GO:0002366
- **Macrophage activation** **GO:0042116**
- **Macrophage activation involved in immune response GO:0002281**
- Mast cell activation GO:0045576
- Mast cell activation involved in immune response GO:0002279
- Mast cell degranulation GO:0043303
- Mast cell mediated immunity GO:0002448
- Microglial cell activation GO:0001774
- Myeloid cell activation involved in immune response GO:0002275
- Myeloid dendritic cell activation GO:0001773
- Myeloid leukocyte mediated immunity GO:0002444
- Neutrophil activation GO:0042119
- Neutrophil mediated cytotoxicity GO:0070942
- Neutrophil mediated immunity GO:0002446
- Neutrophil mediated killing of symbiont cell GO:0070943
- **Regulation of macrophage activation GO:0043030**
- Regulation of mast cell activation GO:0033003
- Regulation of tolerance induction GO:0002643

**Chemokine production**

- CD4-positive, alpha-beta T cell cytokine production GO:0035743
- Positive regulation of cytokine production involved in immune response GO:0002720
- Positive regulation of production of molecular mediator of immune response GO:0002702
- Regulation of cytokine production involved in immune response GO:0002718
- Regulation of production of molecular mediator of immune response GO:0002700
- Regulation of T cell cytokine production GO:0002724
- T cell cytokine production GO:0002369

**TLR and pattern recognition**

- Innate immune response-activating signal transduction GO:0002758
- Pattern recognition receptor signaling pathway GO:0002221
- Positive regulation of innate immune response GO:0045089
- Positive regulation of toll-like receptor signaling pathway GO:0034123
- Regulation of innate immune response GO:0045088
- Regulation of toll-like receptor signaling pathway GO:0034121
- Toll-like receptor signaling pathway GO:0002224

**Fc receptor signaling**

- Fc receptor mediated stimulatory signaling pathway GO:0002431
- Fc-gamma receptor signaling pathway GO:0038094
- Fc-gamma receptor signaling pathway involved in phagocytosis GO:0038096
- Fc receptor signaling pathway GO:0038093
- Immune response-regulating cell surface receptor signaling pathway GO:0002433

involved in phagocytosis

- Regulation of Fc-gamma receptor signaling pathway involved in phagocytosis GO:1905449
- Regulation of Fc receptor mediated stimulatory signaling pathway GO:0060368

**Table S2. Translation of the murine genes of the gene ontology terms (GO) “macrophage migration” (GO:1905517) and “macrophage activation” (GO: 0042116) into the corresponding proteins. Related to Figure 1 and 5.**
Annotations of the indicated GO terms were linked to protein accession IDs which were used to identify the corresponding protein in the protein-database Uniprot. The protein names given by Uniprot were used throughout the manuscript.


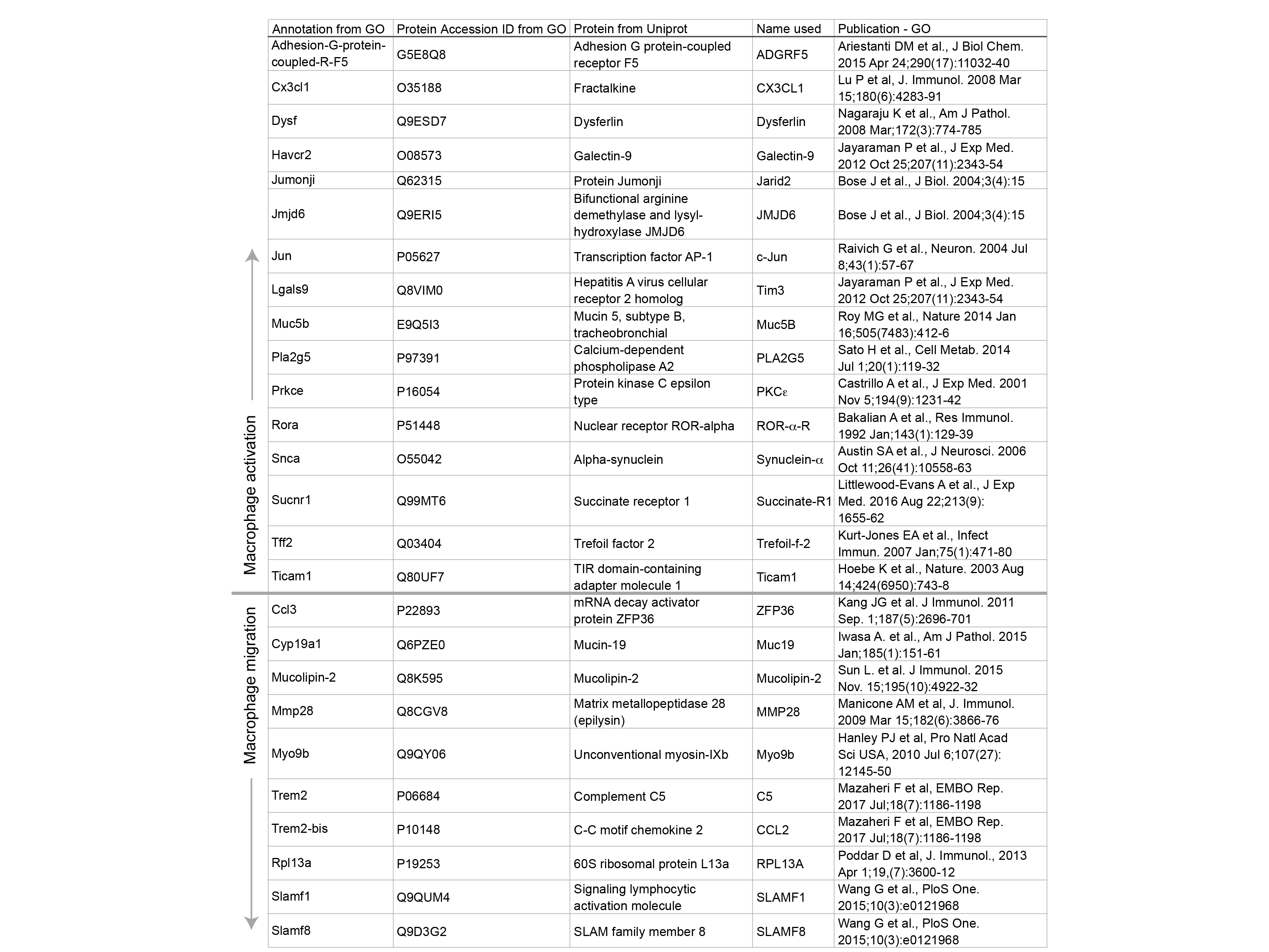


**
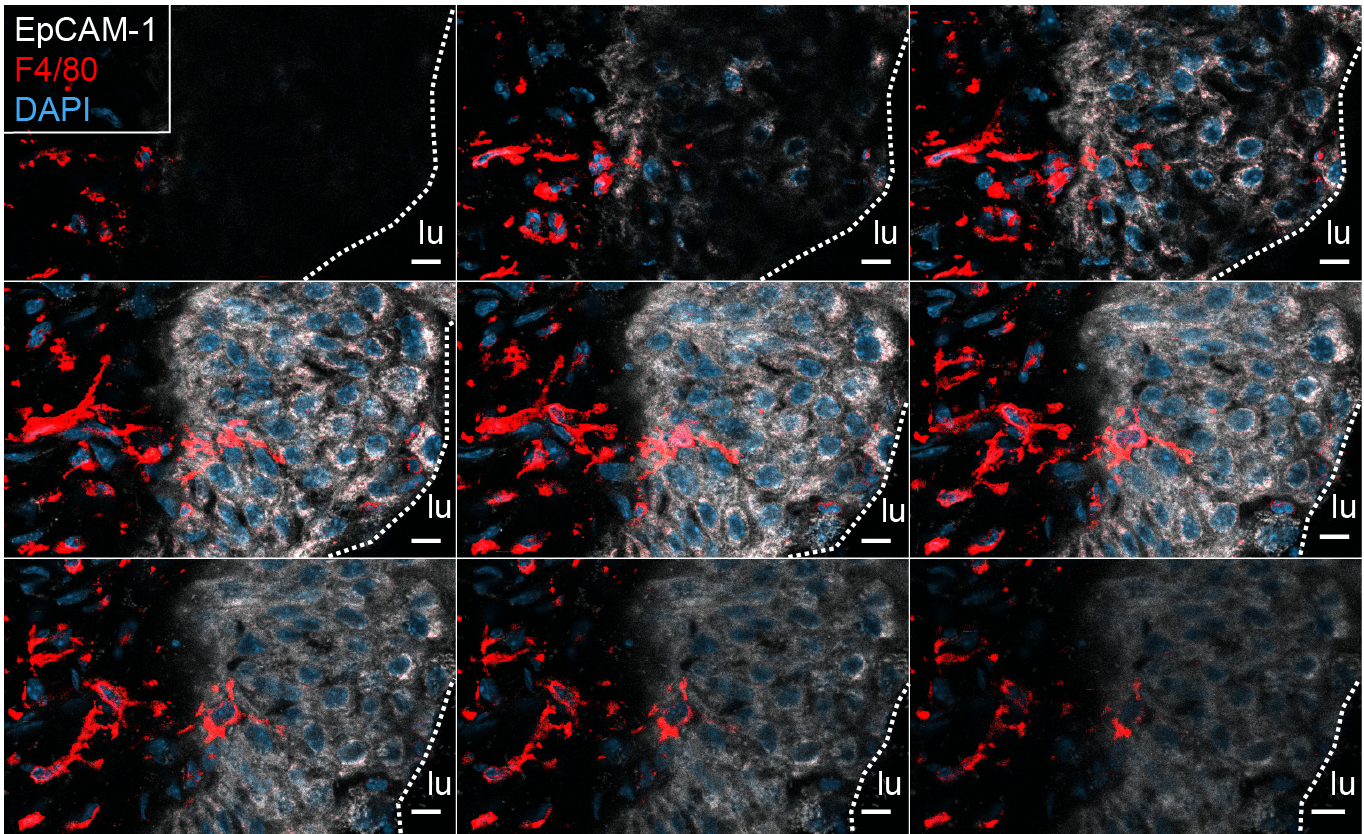
**

**Figure S3. Macrophage relocation into the infected urothelium. Related to Figure 2.**

Mice were infected with UPEC and analyzed one day post infection. Bladder tissue sections were stained with DAPI (blue), F4/80 (red) and EpCAM-1 (white) and imaged by confocal microscopy. The white dashed lines distinguish the urothelium from the connective tissue and lumen. The scale bar indicates 10 µm, step size of the Z-stacks was 0.6 µm. lu=lumen.


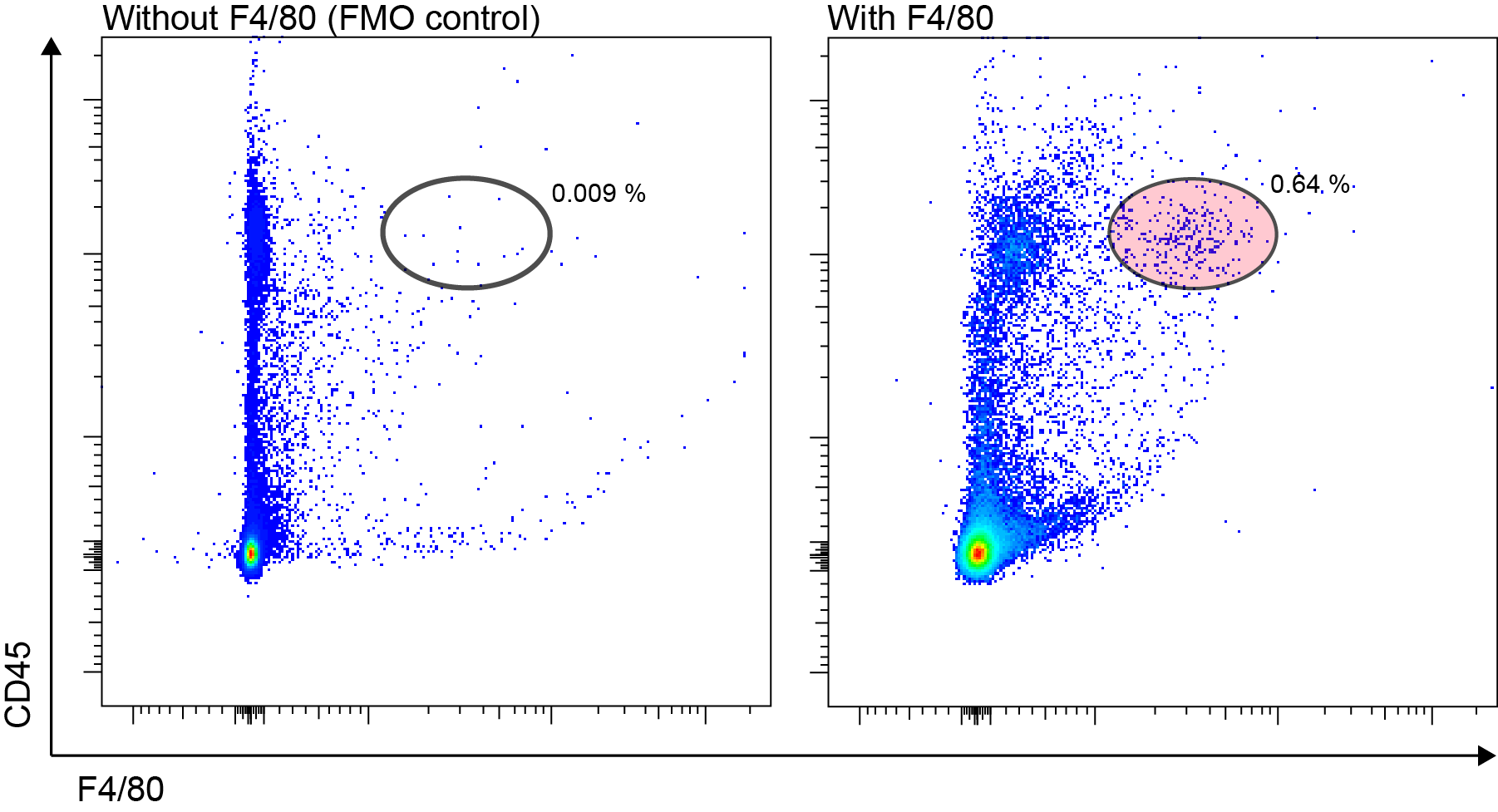


**Figure S4. Gating strategy for macrophages in the urine. Related to Figure 2.**

Flow cytometry gating strategy of CD45^+^F4/80^+^ cells in the urine of UPEC infected mice one day post infection.


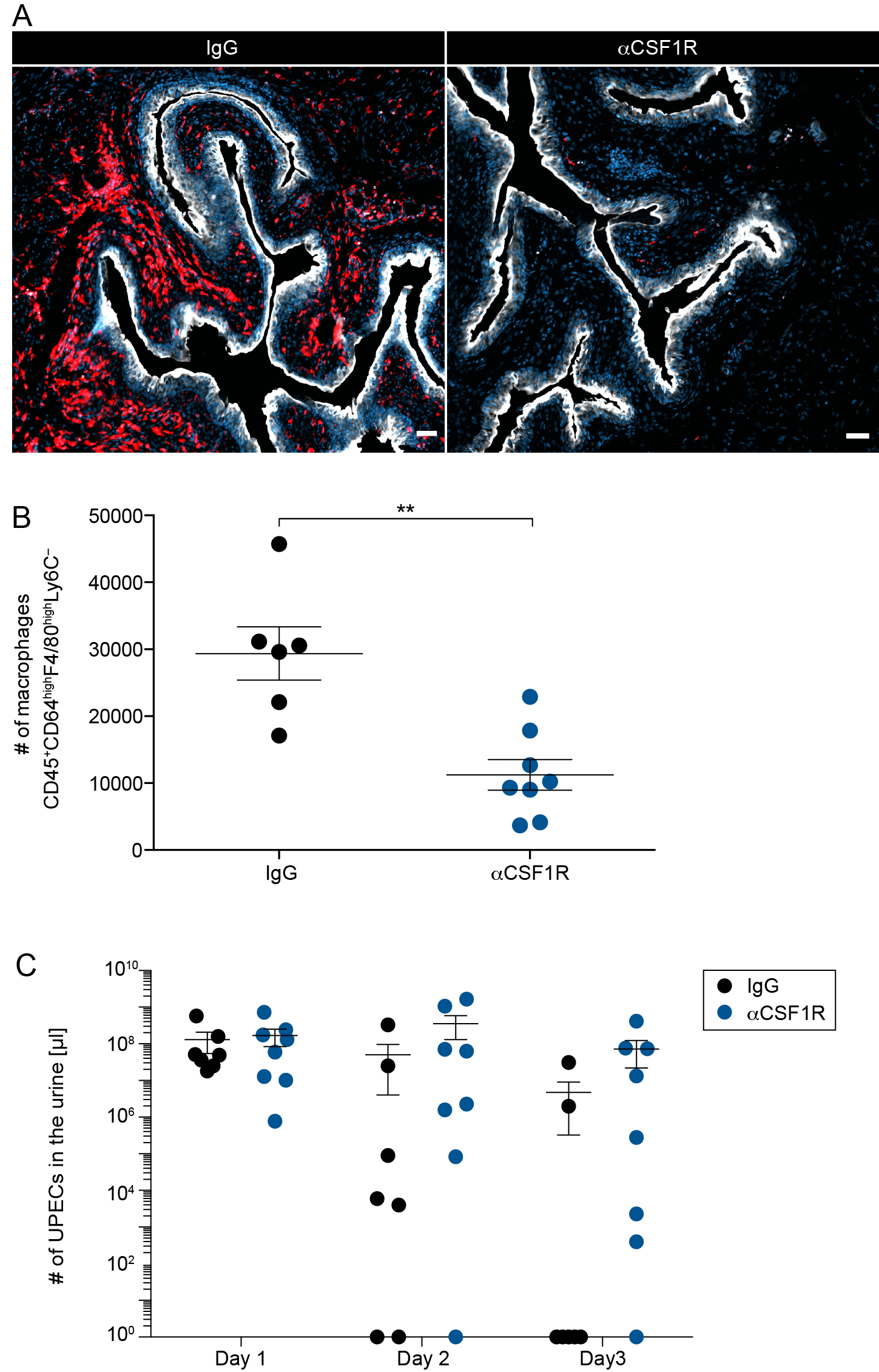


**Figure S5. Macrophage depletion in the urinary bladder. Related to Figure 3.**

Female mice received two intraperitoneal injections of an αCSF1R antibody or isotype control antibodies (IgG).
(**A**) Depletion of macrophages was assessed by immunofluorescence microscopy three days post infection. The scale bar indicates 10 µm. (**B**) Quantification of (A). (**C**) Longitudinal study of the determination of colony forming units (CFU) in the urine of UPEC infected mice in the presence and absence of IL-6.

**p < 0.01. Error bars show the mean ± SEM. The scale bars indicate 50 µm.

**
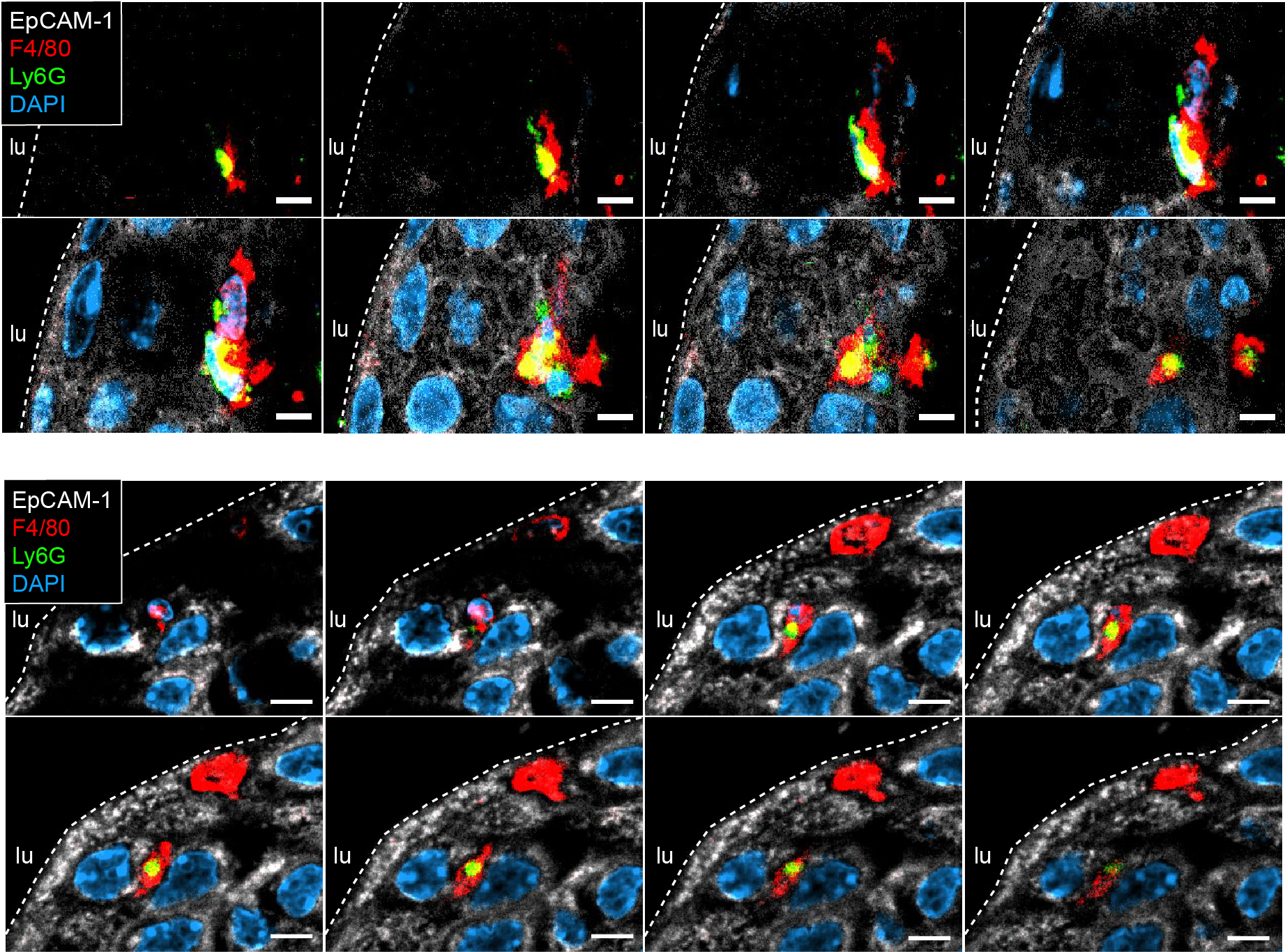
**

**Figure S6. Macrophages phagocytose urothelial neutrophils upon acute bacterial infection of the urinary bladder. Related to Figure 3.**

Mice were infected with UPEC and analyzed one day post infection. Bladder tissue sections were stained with DAPI (blue), F4/80 (red), EpCAM-1 (white) and Ly6G (green) to indicate phagocytosis of neutrophils by macrophages by confocal microscopy. The scale bar indicates 5 µm, step size of the Z‑stacks was 0.4 µm (top) and 0.5 µm (bottom).


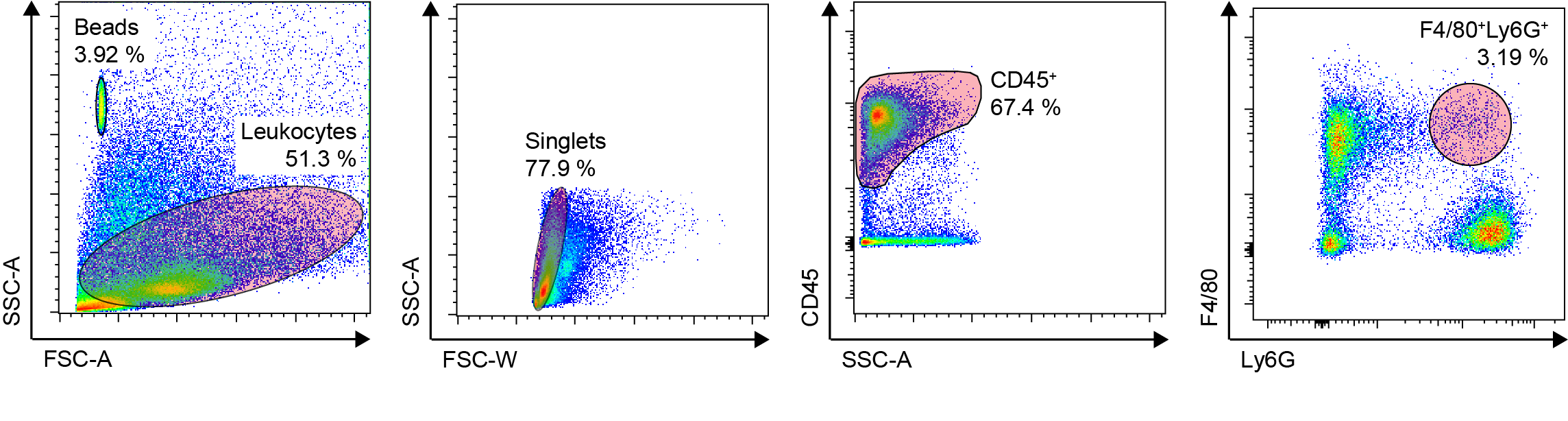


**Figure S7. Gating strategy for macrophage phagocytosis. Related to Figure 3.**

Mice were infected with UPEC and analyzed one day post infection. Representative flow cytometry plots of the uptake of neutrophils by macrophages (CD45^+^F4/80^+^Ly6G^+^).


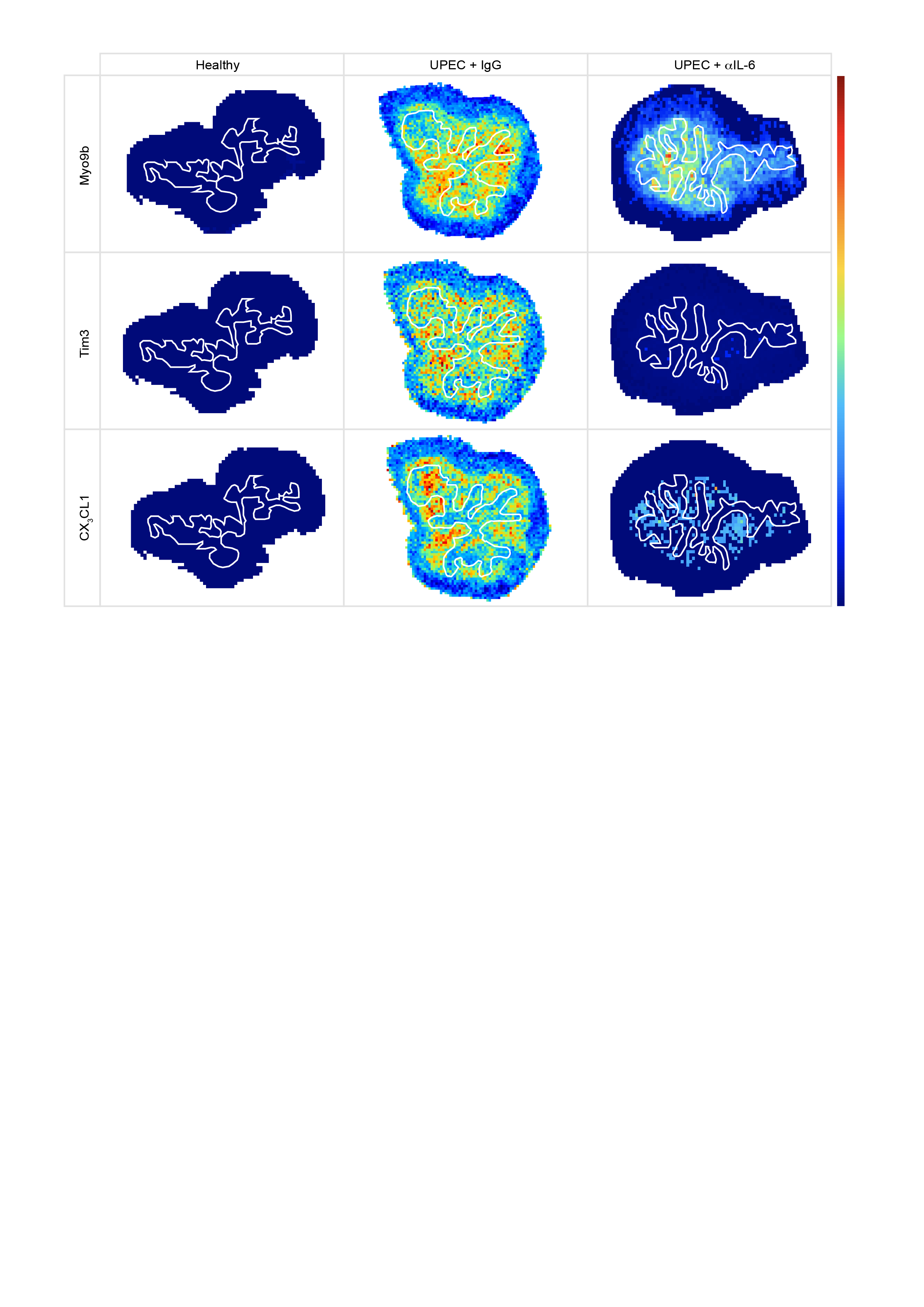


**Figure S8. Spatial expression of molecules involved in macrophage migration determined by MALDI-MSI. Related to Figure 5.**

Mice were infected with UPEC and analyzed one day post infection. Representative spatial distribution of Myo9b, Tim3 and CX_3_CL1 in healthy (left) and UPEC-infected (right) bladders by MALDI-MSI. The top row shows a greyscale image and the tissue segmentation of the urothelium from the urinary bladder. The rows below indicate the expression of the proteins Myo9b, Tim3 and CX_3_CL1. The white lines separate the connective tissue from the urothelium and the lumen. The segmented urothelium and lumen are represented as individual images on the far right and far left sides.
